# Medial swivel dislocation of the talonavicular joint

**DOI:** 10.4103/0019-5413.45329

**Published:** 2009

**Authors:** NS Datt, A Srinivasa Rao, D Venkateswara Rao

**Affiliations:** Department of Orthopaedics, Siddhartha Medical College, Ring Road, Vijayawada - 520 008, Andhra Pradesh, India

**Keywords:** Medial swivel dislocation, subtalar-subluxation, talonavicular dislocation

## Abstract

Medial swivel dislocation, a variant of subtalar dislocation is uncommon. A 35 years old male presented after 6 weeks old injury to left ankle following motor cycle accident. He had pain, swelling around ankle and was unable to bear weight on left foot. Clinical examination revealed diffuse swelling and tenderness in mid foot region. His plain X rays and CT scan showed talonavicular dislocation with compression defect of the head of the talus. He was treated by open reduction and K-wire fixation. At 32 months follow up foot was painless, stable with normal range of ankle and subtalar motion.

## INTRODUCTION

Isolated dislocation of talonavicular joint without associated subtalar joint dislocation or fracture of tarsal bones is rare. These injuries are caused by severe abduction or adduction force applied to the forefoot. Main and Jowett described a variant of subtalar dislocation termed as swivel injury.^1^

A medially or laterally directed force applied to the foot causes dislocation of the talonavicular joint and subluxation but not the dislocation of the subtalar joint. The calcaneum along with remaining foot swivels on the intact interosseous talocalcaneal ligament. A case of medial swivel dislocation treated by open reduction k-wire fixation is reported here.

## CASE REPORT

A 35-year-old male, clerk by occupation, presented with a history of injury to the left foot after a fall while riding a motor cycle six weeks ago. He had pain, swelling, and inability to bear weight on the left foot. Clinical examination revealed diffuse swelling and tenderness in midfoot region. There was no neurovascular compromise in the left foot.

**Figure 1:**
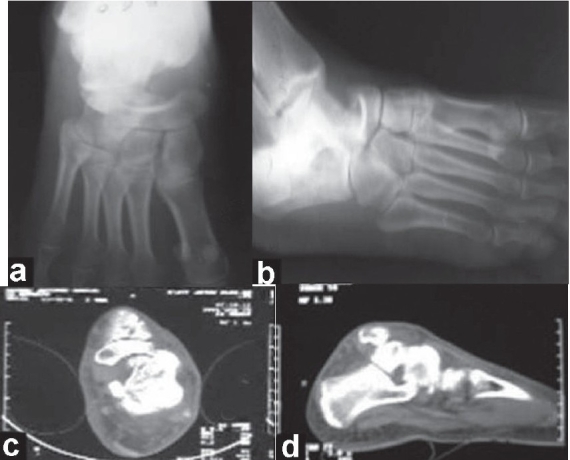
Preoperative radiographs anteroposterior (a); and oblique (b) and CT scan images (c and d) of left foot showing talonanicular dislocation, compression fracture of head of talus, intact calcaneocuboid joint, and a normal subtalar alignment.

Anteroposterior and oblique radiographs of left foot revealed dislocation of talonavicular joint with medial displacement of navicular and compression defect of anteromedial aspect of head of talus [Figure 1a, Figure 1b]. CT scan showed talonavicular dislocation with compression defect of head of talus anteroinferiorly with small fracture fragments and congruent subtalar joint [Figure 1c, Figure 1d].

Under spinal anesthesia, closed reduction was attempted, but it failed. Hence, open reduction was done. Under tourniquet control, a 6 cm anteromedial longitudinal incision was made centering the talonavicular joint. Extensor hallucis longus tendon and dorsalis pedis artery were retracted medially, and extensor digitorum longus tendons were retracted laterally, exposing the dislocated talonavicular joint. The impacted fracture in talus was visualized, which was less than 1 cm size, in all dimensions. The loose fracture fragment was excised.

**Figure 2:**
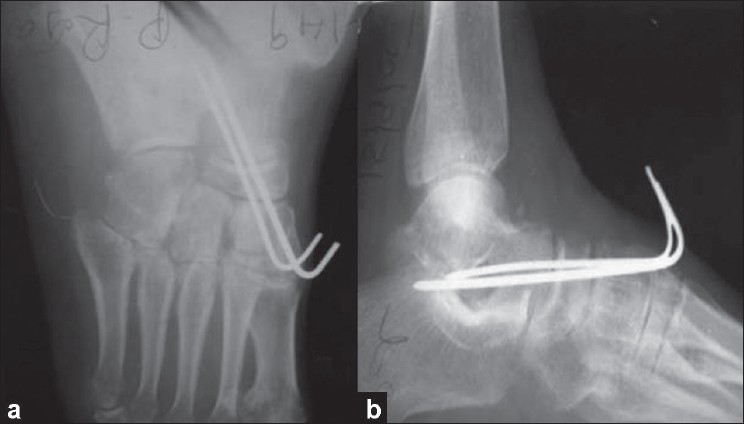
Immediate postoperative anteroposterior (a) and lateral (b) radiographs of left foot showing congruent talanavicular, subtalar, and calcaneocuboid joints with k-wires *in situ*.

The talonavicular joint dislocation was reduced by traction and lateral rotation of the forefoot. The talonavicular joint was stabilized with 2-mm k-wires introduced from the dorsum of the foot transfixing the talonavicular joint [Figure 2]. The wound was closed in layers. The foot was immobilized using below knee cast for 6 weeks. After 6 weeks, k-wires were removed, and weight-bearing was allowed progressively.

**Figure 3:**
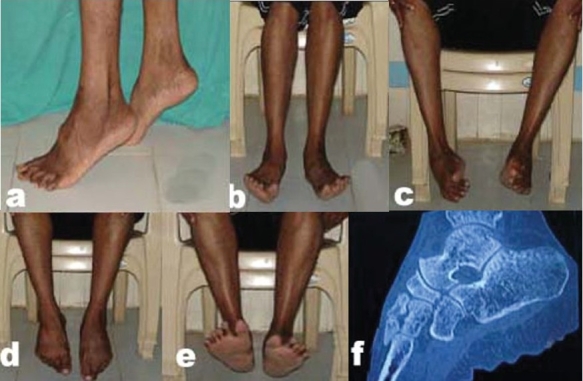
Thirty-two-month follow-up result showing excellent functional (a-e) and radiological outcome. CT scannogram (f), and sagittal CT reconstruction (g) showing congruent subtalar, talanavicular, and calcaneocuboid joints.

After 3 months, the patient returned to his occupation as clerk. The foot was painfree and stable with normal range of ankle and subtalar joint motion. At the recent follow-up of 32 months, the foot was painfree and stable with normal range of ankle and subtalar joint motion [Figure 3a-e]. CT scannogram of left ankle and foot at 32 months showed. The sagittal reconstructed CT films demonstrated the well reduced talonavicular joint and congruent subtalar joint [Figure 3f].

## DISCUSSION

The midtarsal joint includes talonavicular and calcaneocuboid joints, which lie in a transverse plane across foot. Main and Jowett^1^ classified midtarsal injuries according to direction of deforming force and displacement into five groups (medial, longitudinal compression, lateral, plantar, and crush).^1^,^2^ Medial and lateral midtarsal injuries are further divided into fracture, sprain, fracture subluxation or dislocation, and swivel dislocation.^1^

In the swivel injuries, the deforming force applied to the forefoot disrupts talonavicular joint, rotating the foot, causing rotatory subluxation of subtalar joint on the axis of intact interosseous talocalcaneal ligament. In medial swivel injuries, the deforming force is directed medially and talonavicular joint dislocates medially rotating the foot medially, whereas the calcaneocuboid joint is intact. In lateral swivel injuries, the deforming force is directed laterally, dislocating talonavicular joint laterally and rotating foot laterally. It is usually associated with impacted fracture of calcaneocuboid joint (nut cracker fracture).^3^ Swivel injuries differ from subtalar joint dislocation in that the deforming force probably falls more anterior to that which produces subtalar and ankle injuries.^1^,^2^ The talocalcaneal interosseous ligament is intact.

It is important to distinguish between medial subtalar dislocation and swivel dislocation of medial type. Medial subtalar dislocation is reduced by traction and eversion, whereas swivel dislocation is reduced by traction and lateral rotation of foot. Obstruction to closed reduction in subtalar dislocation can be caused by interposed tibialis posterior tendon or interlocked or impacted fracture of articular surfaces of talus/navicular bone, which is not found in swivel type of dislocation.

In the present case, closed reduction was not successful because of late presentation (i.e., after 6 weeks), and hence, open reduction and k-wire fixation was done with good clinical and functional outcome.^4^ This case is presented because of its rarity, unusual mechanism of injury, and excellent outcome of treatment.

## References

[1] Main BJ, Jowett RL (1975). Injuries to the midtarsal joint. J Bone Joint Surg Br.

[2] Rockwood CA, Green DP, Bucholz RW, Heckman JD Fractures in adults.

[3] Pillai A, Chakrabarti D, Hadidi M (2006). Lateral swivel dislocation of the talo-navicular joint. Foot Ankle Surg.

[4] Verhaar JA (1990). Recurrent medial swivel dislocation of the foot. J Bone Joint Surg Br.

